# Computational pharmacology and computational chemistry of 4-hydroxyisoleucine: Physicochemical, pharmacokinetic, and DFT-based approaches

**DOI:** 10.3389/fchem.2023.1145974

**Published:** 2023-04-13

**Authors:** Imad Ahmad, Aleksey E. Kuznetsov, Abdul Saboor Pirzada, Khalaf F. Alsharif, Maria Daglia, Haroon Khan

**Affiliations:** ^1^ Department of Pharmacy, Abdul Wali Khan University Mardan, Mardan, Pakistan; ^2^ Department of Chemistry, Universidad Tecnica Federico Santa Maria, Santiago, Chile; ^3^ Department of Clinical Laboratory, College of Applied Medical Science, Taif University, Taif, Saudi Arabia; ^4^ Department of Pharmacy, University of Naples Federico II, Naples, Italy; ^5^ International Research Centre for Food Nutrition and Safety, Jiangsu University, Zhenjiang, China

**Keywords:** 4-hydroxyisoleucine, pharmacokinetics, computational chemistry, drug likeness, DFT

## Abstract

Computational pharmacology and chemistry of drug-like properties along with pharmacokinetic studies have made it more amenable to decide or predict a potential drug candidate. 4-Hydroxyisoleucine is a pharmacologically active natural product with prominent antidiabetic properties. In this study, ADMETLab 2.0 was used to determine its important drug-related properties. 4-Hydroxyisoleucine is compliant with important drug-like physicochemical properties and pharma giants’ drug-ability rules like Lipinski’s, Pfizer, and GlaxoSmithKline (GSK) rules. Pharmacokinetically, it has been predicted to have satisfactory cell permeability. Blood–brain barrier permeation may add central nervous system (CNS) effects, while a very slight probability of being CYP2C9 substrate exists. None of the well-known toxicities were predicted *in silico*, being congruent with wet lab results, except for a “very slight risk” for respiratory toxicity predicted. The molecule is non ecotoxic as analyzed with common indicators such as bioconcentration and LC_50_ for fathead minnow and *daphnia magna*. The toxicity parameters identified 4-hydroxyisoleucine as non-toxic to androgen receptors, PPAR-γ, mitochondrial membrane receptor, heat shock element, and p53. However, out of seven parameters, not even a single toxicophore was found. The density functional theory (DFT) study provided support to the findings obtained from drug-like property predictions. Hence, it is a very logical approach to proceed further with a detailed pharmacokinetics and drug development process for 4-hydroxyisoleucine.

## 1 Introduction

4-Hydroxyisoleucine (4HIL) is an amino acid which has been purified from *Trigonella foenum-graecum* (fenugreek seeds; Fabaceae). Fenugreek is known for its antidiabetic properties in traditional medicine (Fowden et al., 1973; Abou El-Soud et al., 2007). 4-Hydroxyisoleucine increases insulin secretion *via* a direct effect on isolated islet cells both in rats and humans. The stimulating effect was strictly glucose dependent. Furthermore, it was shown that the insulinotropic effect is biphasic, without associated harm to other islet cells (α and δ-cells) and increase in insulin secretion by increasing glucose concentration (Sauvaire et al., 1998). 4-Hydroxyisoleucine reduces insulin resistance in the muscles and the liver by the activation of insulin receptor substrate-associated phosphoinositide-3 kinase activity. Reduction in body weight and plasma triglyceride and total cholesterol levels resulted from application of 4-hydroxyisoleucine. This plant-derived amino acid offers an attractive package for the treatment of the metabolic syndrome as a whole and also type 2 diabetes, obesity, and dyslipidaemias (Jetté et al., 2000; Narender et al., 2006). Keeping in mind the pharmacological importance of 4HIL, its efficacy needs to be evaluated before it moves to trials and/or is dismissed. Undesirable pharmacokinetic (PK) properties or unacceptable toxicity are the main causes of the failure of drug candidates at the clinical trial stage (Jia et al., 2020).

Since the concept of drug likeness was first proposed, it has become an important consideration in the selection of compounds with desirable bioavailability during the early phases of drug discovery. Over the past decade, online resources have facilitated drug-likeness studies in an economical and time-efficient manner. Michael J. Waring and colleagues compiled and analyzed the attrition of drug candidates from four pharma giants, AstraZeneca, Eli Lilly and Company, GlaxoSmithKline, and Pfizer. They reaffirmed that the control of physicochemical properties during structural optimization helps identify a compound of drug quality. Safety and toxicology are the largest sources of failure within the data set. However, none of the physicochemical descriptors correlate with preclinical toxicology outcomes (Waring et al., 2015).

While considering the various pharmacology effects in different diseased conditions, we calculated various parameters related to drug discovery for 4-hydroxyisoleucine using ADMETLab 2.0. in order to predict the drug-like properties and safety profile and thereby therapeutic potentials.

## 2 Methodology

Various virtual labs are authorized to share the pharmacokinetic profile of pharmacologically active drug candidates such as SwissADME (Daina et al., 2017), FAF-Drugs4 (Lagorce et al., 2017), ADMETlab (Xiong et al., 2021), admetSAR (Yang et al., 2019), pkCSM (Pires et al., 2015), and ProTox-II (Banerjee et al., 2018). However, ADMETLab was selected because it provides predictive spectrum of multiple drug-likeness parameters and most of ADMET properties. The updated version, ADMETLab 2.0, has overpowered all known shortcomings of the older version while keeping its valuable advantages.

This virtual lab calculates 88 ADMET-related properties, i.e., 17 physicochemical parameters, 13 medicinal chemistry properties, 23 ADME properties, 27 toxicity endpoints, and eight toxicophore rules (751 substructures). ADMETLab 2.0 is freely accessible at https://admetmesh.scbdd.com. The 2D structure of 4-hydroxyleucine was drawn in ADMETLab 2.0 (Table 1). The user interface also offers string-based search. The search string for 4-hydroxyisoleucine is CC(O)C(C)C(N)C (=O)O.

**TABLE 1:** Structure, IUPAC name, and SMILES notation of 4-hydroxyisoleucine.

Ligand	4-Hydroxyisoleucine	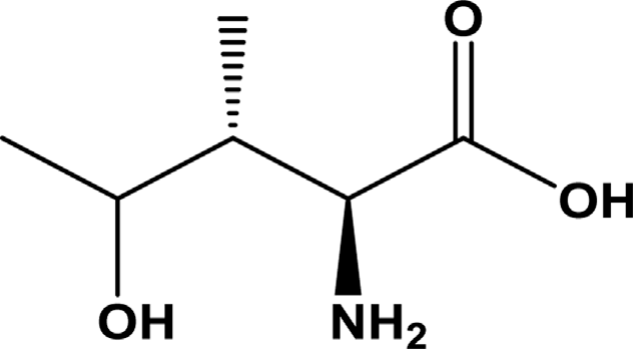
IUPAC	2-Amino-4-hydroxy-3-methylpentanoic acid	
SMILES notation	CC(O)C(C)C(N)C (=O)O	

An oral bioavailability graph obtained from SwissADMET was calculated using physicochemical characteristics. This graph uses six parameters, i.e., lipophilicity, insolubility, size, unsaturation, polarity, and flexibility, and defines appropriate ranges for oral bioavailability. The GI absorption (HIA), BBB penetration, and being Pgp substrate were also predicted with SwissADMET. The result is depicted as “boiled egg” having two areas: one for HIA (white) and another for BBB penetration (yolk). The background is a gray zone, indicating neither GI absorption nor BBB penetration.

The human ether-a-go-go-related gene (hERG) K^+^ channels blockade is linked with fatal cardiac arrhythmias. The hERG K^+^ channel blockade was predicted with both ADMETLab 2.0 and pred-hERG 4.2. The pred-hERG 4.2, http://predherg.labmol.com.br, is a web tool for early detection of presumable hERG blockers and non-blockers, to predict cardiac toxicity.

DFT studies were performed with Gaussian 16 software (Frisch et al., 2016). We optimized the 4-hydroxyisoleucine molecule without any symmetry constraints, checking different possible isomers (*vide infra*), and then performed frequency calculations to verify that the optimized structures were true energy minima. All calculations were performed using the combination of the hybrid density functional B3LYP (Axel, 1993) with the triple zeta split-valence polarized basis set of Ahlrichs and co-workers Def2TZVP (Weigend and Ahlrichs, 2005; Weigend, 2006); the approach is furthermore referred to as B3LYP/Def2TZVP. We studied the 4-hydroxyisoleucine both in the gas phase (vacuum) and with the implicit effects from water (dielectric constant *ε* = 78.3553) as a solvent taken into account. In the second type of calculations, we used the self-reliable IEF-PCM approach (Tomasi et al., 2005) using the UFF default model as implemented in Gaussian 16 software, with the electrostatic scaling factor α = 1.0. The charge analysis was performed using the natural bond orbital (NBO) method as implemented in Gaussian 16 program (Reed et al., 1988), using the B3LYP/Def2TZVP approach with the implicit water effects. Frontier molecular orbitals (FMOs) and molecular electrostatic potential (MEP) were computed at the B3LYP/Def2TZVP level with the implicit water effects as well.

We consider the calculated structures of the seven isomers of 4-hydroxyisoleucine, structural parameters for the lowest-lying isomer, its NBO charges, FMOs, and MEP. Furthermore, we used the values of the energies of the lowest-lying isomer, HOMO and LUMO, to compute its global reactivity parameters (GRP) (Geerlings et al., 2003; Chakraborty et al., 2013; Jorio et al., 2019) (Eqs 1–6). Eqs 1, 2 were used to calculate the values of the ionization potential (*IP*) and electron affinity (*EA*):

$$IP=-E_{HOMO},$$ (1)

$$EA=-E_{LUMO}.$$ (2)

For global hardness *η* and electronegativity *X* values, we used Eqs 3, 4:

$$\eta =\frac{\left[IP-EA\right]}{2}=-\frac{\left[E_{LUMO}-E_{HOMO}\right]}{2}$$ (3)

$$X=\frac{\left[IP-EA\right]}{2}=-\frac{\left[E_{LUMO}-E_{HOMO}\right]}{2}$$ (4)

Also, global electrophilicity *ω* value was calculated by Eq. 5:

$$\omega =\frac{\mu^{2}}{2\eta },$$ (5)

where $\mu =\frac{\left[E_{HOMO}+E_{LUMO}\right.}{2}$ is the chemical potential of the system.

Finally, the global softness *σ* value was computed with Eq. 6:

$$\sigma =\frac{1}{2\eta }$$ (6)

Open GL version of Molden 5.8.2 visualization program was used for the visualization of the structures and FMOs (Schaftenaar and Noordik, 2000), and Avogadro, version 1.1.1, was used to visualize the MEP maps (Hanwell et al., 2012).

## 3 Results

ADMETLab 2.0 offers results in different formats, even in a downloadable pdf format. The following parameters were predicted for 4-HIL.

### 3.1 Physicochemical properties

The widely accepted physicochemical property guidelines are the Lipinski’s rule of five (RO5), which is satisfied by an ideal drug molecule. It offers a prediction of the drug likeness of a chemical compound having a biological activity and designed for oral route of administration. The molecular weight of 4-hydroxyisoleucine is 147.09 g/mol, which reflects its suitability for oral drug development. This provides therapeutic convenience of its formulation as oral dosage form. The optimal range is 100–600 approximately. The molecular volume to van der Waals volume ratio was calculated as 147.06. The number of hydrogen bond acceptors (nHA; optimal: 0–12) is 4. The number of hydrogen bond donors (nHD; optimal: 0∼7) is also 4. 4-Hydroxyisoleucine has three rotatable bonds only (optimal: 0∼11).

There are no rings or cyclic groups in 4-HIL (optimal: 0–6). There are four heteroatoms in 4-hydroxyisoleucine, which satisfy the range 1∼15 given by ADMETLab. There is no formal charge on this molecule. A charge range of −4∼4 is devised as standardized parameter for a drug candidate. The number of rigid bonds may range from 0 to 30; 4HIL has a single rigid bond. This makes the molecule flexible enough to achieve a stable conformation at its target. In this continuity, the flexibility value is 3.0 obtained as ratio of nRot/nRig. The molecule has three stereo centers which is somewhat closer to the optimal value of ≤2. A stereo center may achieve a different conformation at the active site. The topological polar surface area (TPSA) for 4-hydroxyisoleucine is 83.55.

The aqueous solubility (logS) is 0.06, falling within the range of −4∼0.5 log mol/L. LogP value is the Log of octanol/water partition coefficient whose optimal range is 0∼3. A higher logP value implies greater lipophilicity, and this depends upon molecular size, polarity, and hydrogen bonding. LogP value of 4-hydroxyisoleucine is −2.63 which reflects its partition into water compartment. This value is congruent to LogS value described previously. The value of logP at normal physiological pH 7.4 is logD. For 4-hydroxyisoleucine it is calculated as −0.93 just below the optimal range of 1∼3. Physicochemical properties are summarized in Table 2.

**TABLE 2:** Physicochemical properties and medicinal chemistry parameters of 4-hydroxy isoleucine calculated with ADMETLab 2.0.

Physicochemical properties		Medicinal chemistry	
Parameter	Value	Parameter	Value
Molecular weight	147.09 g/mol	QED	0.49
van der Waals volume	147.06	SAscore	3.59
nHA	4	Fsp^3^	0.83
nHD	4	MCE-18	4.0
nRot	3	NP score	0.99
nRing	0	Lipinski rule	Accepted
MaxRing	0	Pfizer rule	Accepted
nHet	4	GSK rule	Accepted
fChar	0	Golden Triangle	Rejected
nRig	1	PAINS	0 alerts
Flexibility = nRot/nRig	3.0	ALARM NMR	0 alerts
Stereo centers	3	BMS	0 alerts
TPSA	83.55 Å^2^	Chelator rule	0 alerts
logS	0.06 log mol/L		
logP	−2.63		
logD	−0.94		

nHA, no. of hydrogen bond acceptors; nHD, no. of hydrogen bond donors; nRot, no. of rotatable bonds; nRing, no. of rings, MaxRing, no. of atoms in the biggest ring; nHet, no. of heteroatoms; fChar, formal charge; nRig, no. of rigid bonds; TPSA, total polar surface area; logS, aqueous solubility; logP, octanol–water partition coefficient; logD, distribution coefficient; QED, quantitative estimate of drug-likeness; SAscore, synthetic accessibility score; Fsp^3^, no. of sp^3^-hybridized carbon/total carbon count; MCE-18, medicinal chemistry evolution; NPscore, natural product-likeness score; GSK, GlaxoSmithKline; and PAINS, pan assay interference compounds.

*Unless otherwise specified, unit of measurement is a number/score explained in the respective text.

### 3.2 Medicinal chemistry

The QED score (quantitative estimate of drug-likeness) is 0.49, which implies it is an unattractive molecule. An attractive drug has a QED score of >0.67; for unattractive molecules, the score is 0.49∼0.67, while it is <0.34 for too complex molecules. The QED score is a drug-likeness score based on the calculated physicochemical properties of marketed oral drugs and published human data.

SAscore is implemented based on synthetic accessibility score, which is an estimated ease of synthesis of a drug-like molecule. Based on SAscore (3.59), 4-hydroxyisoleucine is easy to synthesize. Drug-like molecules having SAscore of ≥6 are difficult to synthesize, while those with a score of <6 are easy to synthesize. The Fsp^3^ value for 4-HIL is 0.83. Fsp^3^ is the fraction or number of sp^3^ carbon atoms out of the total carbon count. This parameter reflects the carbon saturation of molecules and characterizes the complexity of their spatial structure. The suitable value defined by ADMETLab for Fsp^3^ is >0.42, with 4-hydroxyisoleucine being compliant.

The optimum MCE-18 score limit given by ADMETlab 2.0 is ≥45. For 4-HIL MCE-18 score is 4.0, which is extremely low. NP score reflects the natural product-likeness score. This score ranges from −5 to 5; the higher the score, the higher the probability of being a natural product. Natural products are more likely to be safe, with reduced risk of toxicity. NP score for 4-hydroxyisoleucine is 0.99, which is in this safe range.

ADMETLab 2.0 has described Lipinski’s rule as follows: MW ≤ 500, logP ≤5, Hacc ≤10, and Hdon ≤5. These parameters are already described individually under physicochemical properties. All these parameters are met by 4-HIL and thus the widely accepted Lipinski rule is satisfied. In the case where two of these properties are out of range, there is a possibility of poor absorption or permeability. Deviation of only one property is allowed. 4-Hydroxyisoleucine also complies with Pfizer’s rule, i.e., logP >3 and TPSA <75. Compounds which satisfy the GSK rule may have a more favorable ADMET profile. 4-HIL concurs with GSK’s rule as well. The Golden Triangle rule devises the range for two parameters as follows: 200 ≤ MW ≤ 50 and −2 ≤ logD ≤5. Compounds satisfying this Golden Triangle rule are more likely to have favorable ADMET profiles. As per this criterion, 4-hydroxyisoleucine is rejected. However, ADMET profiles are separately described later which possess desirable pharmacokinetics.

4-HIL has zero PAINS alerts, and thus has been excluded from frequent hitters, α-screen artifacts, and reactive compounds. 4-Hydroxyisoleucine has no ALARM NMR alerts and is not a thiol reactive compound. 4-Hydroxyisoleucine has no red alert for BMS and the chelator rule and so is a desirable, non-reactive, and non-chelating compound. Medicinal chemistry parameters are summarized in Table 2.

### 3.3 Pharmacokinetics

#### 3.3.1 Absorption

Caco-2 cell lines (Caucasian colon adenocarcinoma) are still widely used in absorption studies. The Caco-2 permeability score obtained was −5.99 which is lower than the minimum optimal score of −5.15 log unit. The Madin–Darby canine kidney (MDCK) permeability score was 0.01 cm/s which is in the acceptable limit. The standard scores for low permeability, medium permeability, and high passive permeability are <2 × 10^−6^ cm/s, 2–20 × 10^−6^ cm/s, and >20 × 10^−6^ cm/s, respectively.

4-Hydroxyisoleucine has a very low probability as Pgp inhibitor as per its score of 0.01. Therefore, it is regarded as safe from having significant drug interactions. For an inhibitor, the score is 1, while 0 score denotes a non-inhibitor. This output value exhibits the probability of being a Pgp inhibitor. The probability of being Pgp substrate is the lowest, as indicated by its score 0.05. For a Pgp substrate, the score is 1, while for a non-substrate, the score is 0.

4-HIL is predicted to have low human intestinal absorption (HIA <30%) as indicated by its HIA score, 0.09. The bioavailability is predicted to be greater than 20% and 30% (F_20%_ 0.004, F_30%_ 0.002).

**FIGURE 1:**
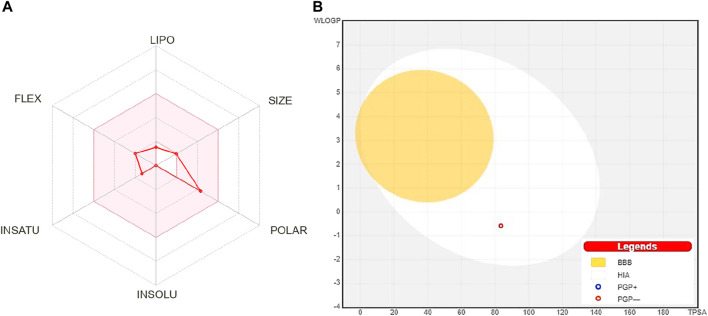
**(A)** Oral bioavailability graph from the SwissADME database; the pink zone is the physicochemical space for oral bioavailability, and the red line defines oral bioavailability properties of 4-hydroxyisoleucine. **(B)** Predicted boiled-egg plot from the SwissADME database reveals human intestinal absorption and not being Pgp substrate. LIPO: lipophilicity, POLAR: polarity, INSOLU: insolubility, INSATU: unsaturation, FLEX: flexibility, BBB: blood–brain barrier, HIA: human intestinal absorption, PGP+: p-glycoprotein substrate, and PGP-: not a p-glycoprotein substrate.

The oral bioavailability graph shown in Figure 1A is predicted with the SwissADMET database. This graph is based on six physicochemical characteristics, i.e., lipophilicity, size, polarity, insolubility, unsaturation, and flexibility. The result of the compound 4-hydroxyisoleucine was within these limits, demonstrating a favorable physio-chemical profile, which is a prerequisite factor that must be monitored in pharmaceutical and clinical studies. The colored zone is the suitable physicochemical space for oral bioavailability.

HIA and central nervous system (CNS) absorption needs to be checked for every biomolecule before its formulation in the pharmaceutical or clinical trials. The blood–brain barrier penetration is essential only if the compound has a target in the CNS, but the inactive compounds on the CNS must not intersect to avoid adverse effects. The boiled-egg plot shows that 4-hydroxyisoleucine has a high gastrointestinal absorption without BBB permeability, indicating low occurrence for CNS side effects (Figure 1B).

#### 3.3.2 Distribution

Plasma protein binding (PPB), unbound fraction in plasma (Fu), volume of distribution (VD), and blood–brain barrier (BBB) permeability were considered to study the distribution (Table 4). The predicted PPB was 10.90%. The optimal range of PPB is <90% because drugs which are high protein-bound may have a low therapeutic index. The volume of distribution (VD) was calculated as 0.39 L/kg. The optimal range for VD is 0.04–20 L/kg. 4-Hydroxyisoleucine has more tendency for blood–brain barrier penetration reflected by the score, 0.73. The output value indicates the probability of being able to cross BBB. The unbound fraction in plasma (Fu) was calculated as 87.84%. The scores for low, middle, and high unbound plasma fraction are <5%, 5∼20%, and >20%, respectively. This implies that more unbound plasma fraction will be available for pharmacological action.

#### 3.3.3 Metabolism

This parameter is important from the drug plasma concentration perspective. The database defines the ligands’ probability to inhibit the enzyme or not by its placement either in category 1 (Inhibitor) or category 0 (non-inhibitor). Similarly, the probability of being substrate for the enzyme is exhibited by the score 1 or 0. Category 1 denotes the molecule is substrate, while category 0 describes it to be non-substrate of the enzyme.

4-Hydroxyisoleucine is likely a non-inhibitor of CYP1A2 as indicated by the assigned score 0.01. The CYP1A2 substrate score obtained as 0.06 implies it is a non-substrate. The probability of CYP2C19 inhibition and being CYP2C19 substrate is extremely low. 4-Hydroxyisoleucine appeared to be non-inhibitor of CYP2C9. However, very low probability exists for being CYP2C9 substrate. Similarly, there is no incidence of CYP2D6 inhibition and being a CYP2D6 substrate. 4-Hydroxyisoleucine is not deemed a CYP3A4 inhibitor nor is it a substrate for CYP3A4.

#### 3.3.4 Excretion

The clearance (CL) for 4-hydroxyisoleucine is calculated as 7.27 mL/min/kg which is a moderate clearance rate. If a drug has a high clearance rate, its score will be > 15 mL/min/kg; for a moderate clearance rate, it will be 5–15 mL/min/kg; and for a low clearance rate, it will be < 5 mL/min/kg. The half-life (T_1/2_) value is 0.62, which reflects the probability of long half-life. The database output value may be from category 1 (long half-life, >3 h) or category 0 (short half-life, <3 h). All the pharmacokinetic parameters (absorption, distribution, metabolism, and excretion) are summarized in Table 3.

**TABLE 3:** Pharmacokinetics of 4-Hydroxy isoleucine calculated with ADMETLab 2.0.

Parameter	Value/probability	Parameter	Value/probability
Absorption		Metabolism	
Caco-2 permeability	−5.99	CYP1A2 inhibitor	0.01
MDCK permeability	0.01 cm/s	CYP1A2 substrate	0.06
Pgp inhibitor	0.01	CYP2C19 inhibitor	0.038
Pgp substrate	0.05	CYP2C19 substrate	0.10
HIA	0.09	CYP2C9 inhibitor	0.02
F_20%_	0.004	CYP2C9 substrate	0.49
F_30%_	0.002	CYP2D6 inhibitor	0.04
Distribution		CYP2D6 substrate	0.19
PPB	6.73%	CYP3A4 inhibitor	0.01
VD	0.46 L/kg	CYP3A4 substrate	0.07
BBB penetration	0.83	Excretion	
Fu	85.29%	CL	7.26 mL/min/kg
		T_1/2_	0.68

Caco-2, Caucasian colon adenocarcinoma cell lines; MDCK, Madin–Darby canine kidney; Pgp, *para*-glycoprotein; HIA, human intestinal absorption; F, bioavailability; PPB, plasma protein binding; VD, volume of distribution; BBB, blood–brain barrier; Fu, unbound fraction in plasma; CYP, cytochrome P450; CL, clearance; T_1/2_, half-life. *Unless otherwise specified, unit of measurement is a probable score explained in the respective text.

### 3.4 Toxicity

The toxicity parameters verified by ADMETlab include hERG blockade, human hepatotoxicity, drug-induced liver injury, AMES toxicity, rat oral acute toxicity, FDA maximum daily dose, carcinogenicity, mutagenicity, skin sensitization, eye corrosion, eye irritation, and respiratory toxicity (Table 4).

**TABLE 4:** Toxicity profile of 4-hydroxy isoleucine calculated with ADMETLab 2.0.

Parameter	Probability
hERG blockers	0.01
H-HT	0.11
DILI	0.02
AMES toxicity	0.01
Rat oral acute toxicity	0.11
FDAMDD	0.01
Skin sensitization	0.11
Carcinogenicity	0.03
Eye corrosion	0.02
Eye irritation	0.07
Respiratory toxicity	0.45

hERG, human ether-a-go-go-related gene; H-HT, human hepatotoxicity; DILI, drug-induced liver injury; FDAMDD, FDA maximum daily dose.

*Unless otherwise specified, the unit of measurement is a probable score explained in the respective text.

**FIGURE 2:**
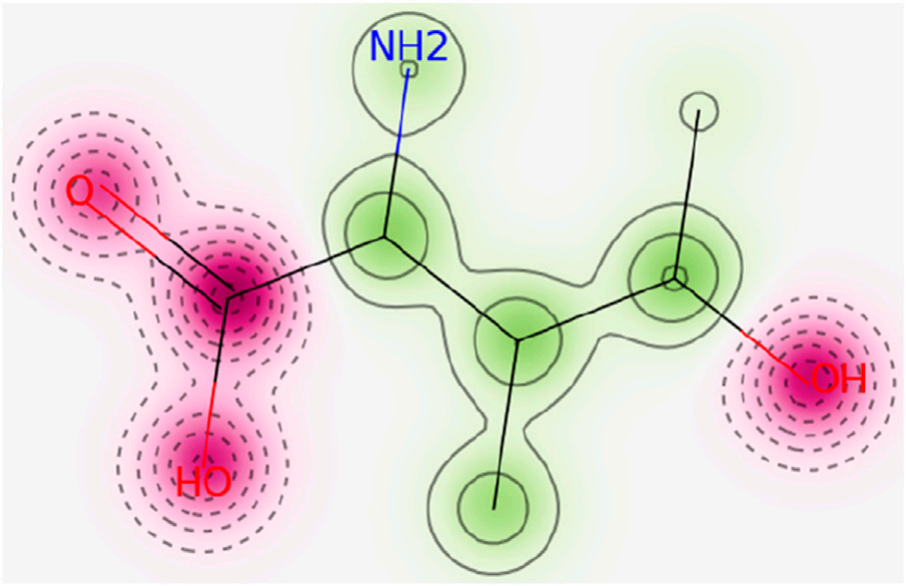
In the probability map of hERG blockage (Pred-hERG web tool), the red dotted lines reflect non-hERG blocker. Contour lines with intense green color indicate a higher positive contribution of an atom to hERG blockade.

The ADMETLab 2.0 score for hERG blocker is 0.01 which shows the safety of 4-hydroxyisoleucine. The result was further in line with Pred-hERG which defined 4-hydroxyisoleucine as non-cardiotoxic with 90% confidence, as shown in Figure 2. The red dotted lines defined the area which negatively contributes to hERG blockade, while contour lines with intense green color represent a higher positive contribution of an atom to hERG blockade. 4-Hydroxyisoleucine is not human hepatotoxic (H-HT) as its score is 0.11. Molecules falling in category 1 are hepatotoxic (H-HT positive, +), while category 0 reflects non-hepatotoxic (H-HT negative, −). Similarly, there is low risk of drug-induced liver injury (DILI), 0.02. Commonly, category 1 drugs have high risk of DILI, while category 0 drugs have no risk of DILI. Furthermore, Ames toxicity score also indicated safety of 4-hydroxyisoleucine, evident from its score of 0.01. Category 1 drugs are classified as Ames positive (+, toxic), while category 0 represents Ames negative (−, non-toxic). Finally, the risk of oral toxicity is low. The rat oral acute toxicity score is 0.11, which classifies 4-hydroxyisoleucine in category 0 (low-toxicity), as category 1 indicates high-toxicity.

4-Hydroxyisoleucine has the probability of being skin non-sensitizer with a value of 0.11. Category 1 drugs are skin sensitizers, while category 0 are non-sensitizers. The risk of carcinogenicity (0.03) is lower, and the molecule is safe from adverse reactions. Category 1 drugs are carcinogens, while category 0 are non-carcinogens. A negligible risk of eye corrosion is predicted as exhibited by its score, 0.02. Category 1 drugs are corrosives, while category 0 drugs are non-corrosive to the eye. The output value indicates the probability of being non-irritant to the eye. The eye irritation score is 0.07. Category 1 drugs are irritants, while category 0 drugs are non-irritants. Finally, the respiratory toxicity score (0.45) declares borderline low probability to be free from respiratory toxicity. Category 1 indicates respiratory toxicants, and category 0 drugs are believed to be free from respiratory toxicity.

### 3.5 Environmental toxicity

For a drug, it is also important to be non-toxic to the environment. Therefore, ADMETLab defines some of the environmental toxicity indicators which are provided in Table 5. The bioconcentration factor, 0.17, is in favor of 4-hydroxyisoleucine non-ecotoxicity. The 50% growth inhibitory concentration (IGC_50_) for *Tetrahymena pyriformis* is 2.43. For 96-h fathead minnow (*Pimephales promelas*), the 50% lethal concentration LC_50_FM of 4-hydroxyisoleucine is 2.92 (Dionne et al., 2021). The score for a 48-h *daphnia magna* 50 percent lethal concentration (LC_50_DM) for 4-hydroxyisoleucine is 2.36.

**TABLE 5:** Environmental toxicity of 4-hydroxyisoleucine calculated with ADMETLab 2.0.

Parameter	Value
Bioconcentration factors	0.167-log10 [(mg/L)/(1,000*MW)]
IGC_50_ *Tetrahymena pyriformis*	2.432 -log10 [(mg/L)/(1,000*MW)]
LC_50_FM (fathead minnow)	2.916 -log10 [(mg/L)/(1,000*MW)]
LC_50_DM *daphnia magna*	3.199 -log10 [(mg/L)/(1,000*MW)]

IGC_50_, 50% growth inhibitory concentration and LC_50_, 50% lethal concentration.

### 3.6 Tox21 pathway

There is probability, to a negligible extent, of 4-hydroxyisoleucine binding to androgen receptor (NR-AR). The output value is 0.05, indicating its safety toward genetic instability. In another approach to test its safety, it is predicted that 4-hydroxyisoleucine will not bind to androgen receptor ligand-binding domain (NR-AR-LBD). Such a reduced probability is exhibited from its lowest value 0.01.

With such a reduced probability (0.01), inactivity at aryl hydrocarbon receptor is evidenced. No activity at NR-Aromatase is predicted, as 0.01 is near to zero, which means inactive. However, 4-hydroxyisoleucine has a low probability to remain inactive at binding to the estrogen receptor (0.42), while reduced affinity for estrogen receptor ligand-binding domain exists (0.01). The peroxisome proliferator-activated receptor gamma is also unlikely to be affected by 4-hydroxyisoleucine, as the score is 0.01. 4-Hydroxyisoleucine is not predicted to be an antioxidant response element since the probability is negligible at 0.02. It is also unlikely to interfere with the activity of ATPase family AAA domain-containing protein 5 as predicted score is 0.01. Furthermore, it is unlikely to activate the heat shock factor response element as the score is 0.01. The score for non-modification of mitochondrial membrane potential is 0.01. Finally, 4-hydroxyisoleucine also has low probability (0.01) of affecting p53 (see Table 6).

**TABLE 6:** Toxicity profile of 4-hydroxyisoleucine calculated with ADMETLab 2.0.

Parameter	Probability
NR-AR	0.05
NR-AR-LBD	0.01
NR-AhR	0.01
NR-Aromatase	0.01
NR-ER	0.42
NR-ER-LBD	0.01
NR-PPAR gamma	0.01
SR-ARE	0.02
SR-ATAD5	0.01
SR-HSE	0.01
SR-MMP	0.01
SR-p53	0.01

NR-AR, androgen receptor; NR-AR-LBD, androgen receptor ligand-binding domain; NR-AhR, aryl hydrocarbon receptor; NR-ER, estrogen receptor, NR-ER-LBD, estrogen receptor ligand-binding domain; NR-PPAR gamma, peroxisome proliferator-activated receptor gamma; SR-ARE, antioxidant response element; SR-ATAD5, ATPase family AAA domain-containing protein 5; SR-HSE, heat shock factor response element; SR-MMP, mitochondrial membrane potential.

### 3.7 Toxicophore rules

This parameter rules out the presence of a toxicophore, causing severe adverse effects. The acute toxicity rule exhibit zero alerts for acute toxicity during oral administration. The molecule is safe from genotoxic carcinogenicity or mutagenicity. Furthermore, 4-hydroxyisoleucine is clear of a non-genotoxic carcinogenic mechanism as well, with zero alerts. No toxicophore was found for skin sensitization, and 4-hydroxyisoleucine is expected to be free from skin irritation. The molecule is not toxic to aquatic life, with zero alerts for the aquatic toxicity rule (water toxicity). 4-Hydroxyisoleucine also appears biodegradable as ADMETlab revealed zero alerts for the non-biodegradable rule. 4-Hydroxyisoleucine has no enlistment in SureChEMBL database, so it has zero alerts for the SureChEMBL rule and is not classified as MedChem unfriendly status (see Table 7).

**TABLE 7:** Toxicophore based profile of 4-hydroxy isoleucine calculated with ADMETLab 2.0.

Parameter	Value
Acute toxicity rule	0 alerts
Genotoxic carcinogenicity rule	0 alerts
Non-genotoxic carcinogenicity rule	0 alerts
Skin sensitization rule	0 alerts
Aquatic toxicity rule	0 alerts
Non-biodegradable rule	0 alerts
SureChEMBL rule	0 alerts

### 3.8 DFT study results

**FIGURE 3:**
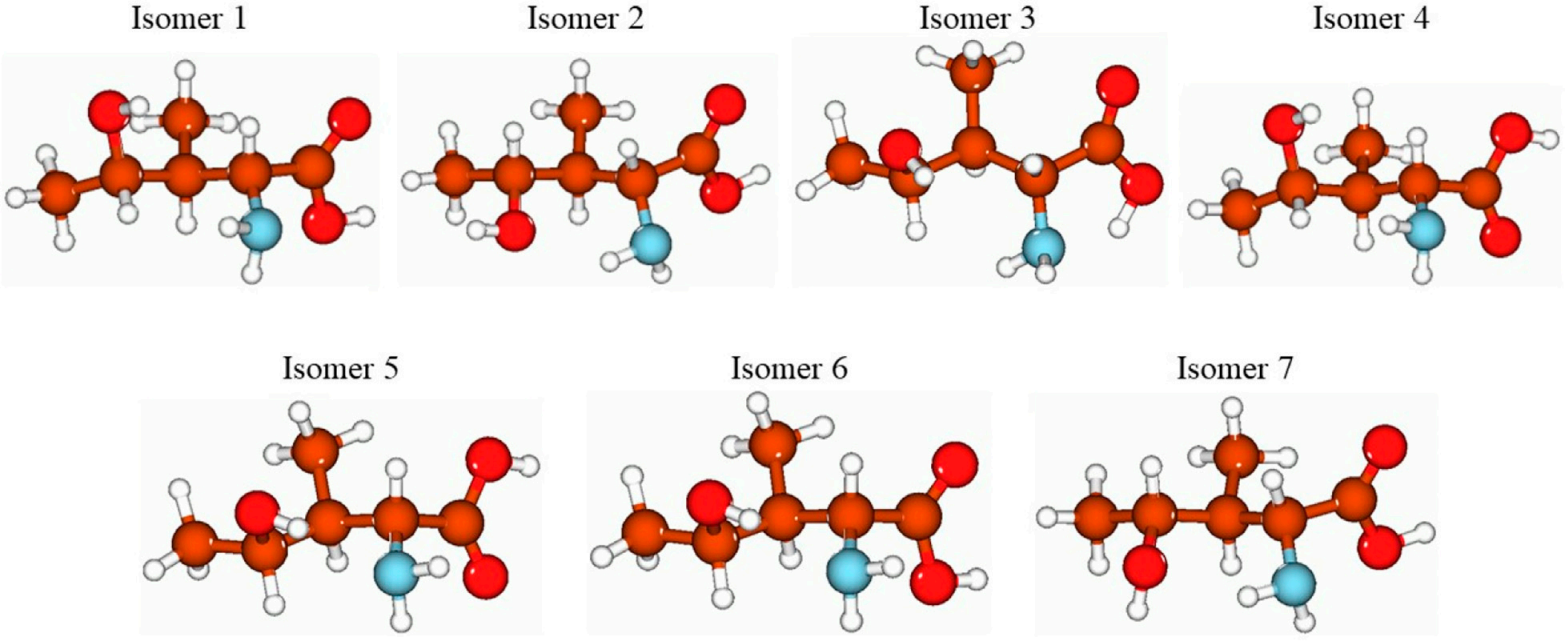
Structures of the seven studied isomers of the 4-hydroxyisoleucine optimized using the B3LYP/Def2TZVP method with the implicit effects of water. Color coding: brown for C, red for O, light blue for N, and light gray for H.

In Figure 3, the optimized structures of seven isomers studied for 4-hydroxyisoleucine are shown, and Table 8 summarizes the energies, relative energies, HOMO/LUMO energies, and HOMO/LUMO energy gaps of these seven isomers. As can be seen from Figure 3, the isomers differ from each other by orientations of 4-hydroxy and amino groups and hydrogens of the carbonyl, hydroxyl, and amino groups. The lowest-lying isomer 5 (Figure 3) has the carbonyl oxygen of the carboxyl group and amino group located on the same molecule side and the 4-hydroxy group located in the plane approximately perpendicular to the carboxyl group, with hydrogen bonds formed between the 4-hydroxy and amino groups and between the amino group and the carbonyl oxygen of the carboxyl group (see more detailed discussion later). Furthermore, from Table 8, it follows that all six higher-lying 4-hydroxyisoleucine isomers are quite close in energy to the global minimum isomer 5, being within 0.09 (isomer 3)–2.89 (isomer 7) kcal/mol higher in energy. We focus on the more detailed consideration of only the lowest-lying isomer 5. Further consideration of Table 4 data shows that all seven isomers have significant HOMO/LUMO gaps, 6.51–7.07 eV, with the isomer 5 having the HOMO/LUMO gap value 6.86 eV, the highest in energy isomer 7 having the smallest HOMO/LUMO gap value, 6.51 eV, and the second lowest-lying isomer 3 having the largest HOMO/LUMO gap value, 7.07 eV.

**TABLE 8:** Energetics of the seven optimized isomers of 4-hydroxyisoleucine calculated using the B3LYP/Def2TZVP method with the implicit effects of water.

Isomer	E_0_A.U.	E_0_ + ZPEA.U.	DE, kcal/mol	E (HOMO/LUMO)	DE (HOMO/LUMO), eV
				A.U.	
1	−517.127629	−516.931358	2.51	−0.25518/-0.00954	6.68
2	−517.127409	−516.930878	2.65	−0.25034/-0.00949	6.55
3	−517.131486	−516.934972	0.09	−0.26491/-0.00496	7.07
4	−517.128038	−516.931992	2.25	−0.25778/-0.01037	6.73
5	−517.131629	−516.934014	0.0	−0.26509/-0.01290	6.86
6	−517.130944	−516.933490	0.43	−0.25819/-0.01357	6.66
7	−517.127028	−516.930205	2.89	−0.24850/-0.00925	6.51

**FIGURE 4:**
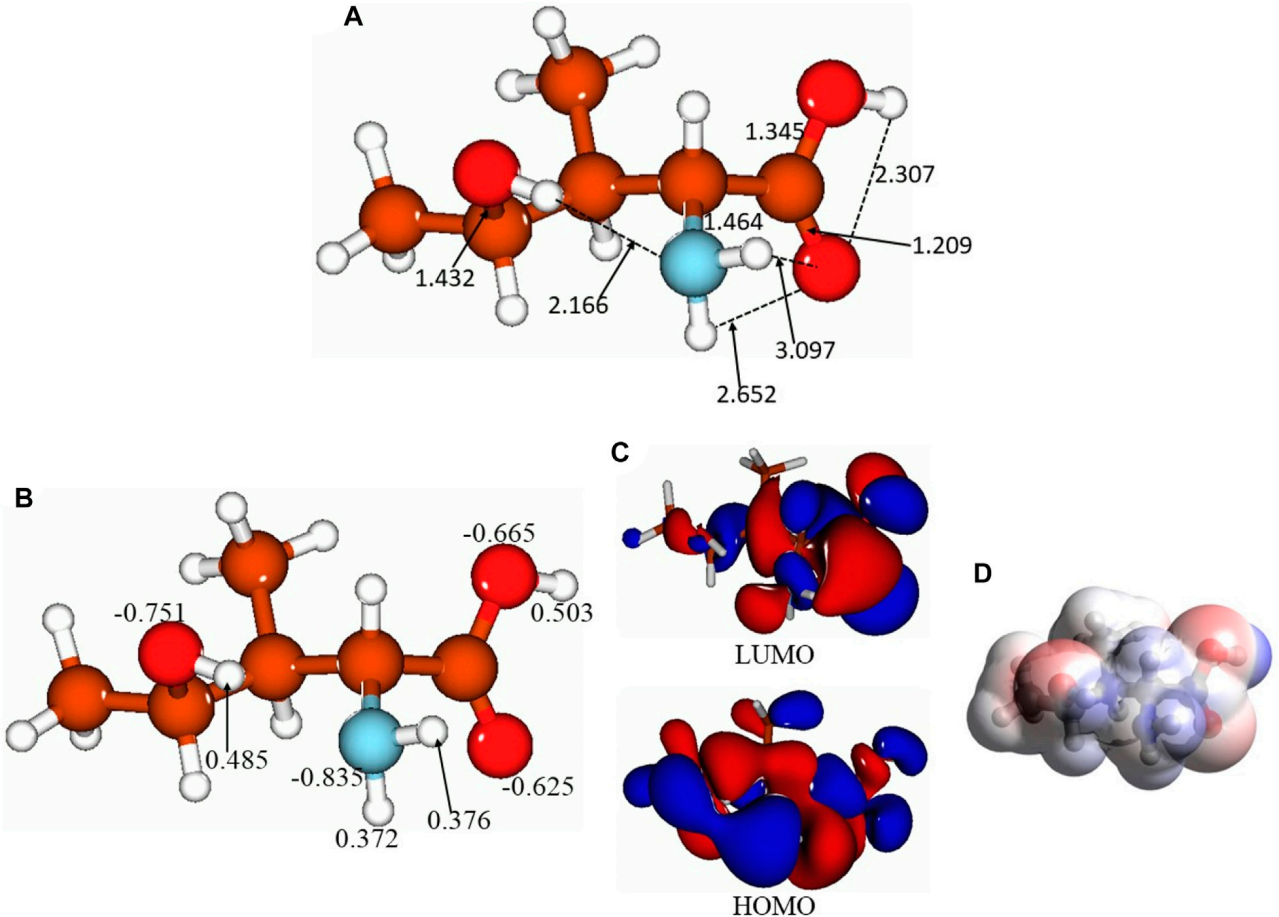
Lowest-lying isomer 5 of 4-hydroxyisoleucine: selected bond distances, Å **(A)**, NBO charges on the selected atoms **(B)**, FMOs **(C)**, and MEP plot **(D)**.

Figure 4 presents the selected bond distances, Å (Figure 4A), NBO charges on the selected atoms (Figure 4B), FMOs (Figure 4C), and MEP plot (Figure 4D) for the lowest-lying isomer 5. As aforementioned, there are several intermolecular hydrogen bonds in this isomer, between the 4-hydroxy group hydrogen and amino group nitrogen (bond distance, 2.166 Å), between the amino group hydrogens and the carbonyl oxygen of the carboxyl group (bond distances, 2.652 and 3.097 Å), and within the carboxyl group, 2.307 Å. Thus, as can be seen, intra- and also intermolecular hydrogen bonds can be formed by 4-hydroxyisoleucine, which might explain the presence of several isomers close in energy. Furthermore, NBO charge analysis (Figure 4B) shows noticeable negative charges on the oxygens and nitrogen of the molecular functional groups, −0.625 to −0.835 e, along with noticeable positive charges on the hydrogens of these functional groups, 0.372–0.503 e. These charges would explain the formation of intramolecular hydrogen bonds along with intermolecular interactions. The HOMO and LUMO plots (Figure 4C) show that essentially the whole molecule contributes to FMOs, thus being able to participate in potential processes of donating/accepting electrons. Finally, analysis of the MEP plot (Figure 4D) shows accumulation of both negative (as indicated by red color) and positive (as indicated by blue color) electrostatic potential in the molecule. Negative MEP is accumulated on the carboxyl oxygens and on the 4-hydroxy oxygen, whereas positive MEP is accumulated on the amino group, carboxyl group hydrogen, and 4-hydroxy group hydrogen. This implies that the 4-hydroxyisoleucine molecule can behave both as a nucleophilic and electrophilic agent in chemical reactions. Also, these MEP accumulations imply possibility of intermolecular interactions for this compound.

Table 8 summarizes the values of the GRPs calculated according to Eqs 1–6 (see Materials and Methods section).

Analysis of the GRP values in Table 9 shows the following. 1) The 4-hydroxyisoleucine compound has quite a high IP value, 7.21 eV, but low EA value, 0.35 eV. This implies that this compound should be a poor electron donor and a relatively poor electron acceptor, or, in other words, should be relatively stable in redox processes, which is also supported by its significant HOMO/LUMO gap value, 6.86 eV. 2) The global electronegativity *X* and global hardness *η* values of this compound are quite noticeable, 3.78 and 3.43 eV, respectively, which would imply that this compound might show electrophilic behavior in chemical processes but also should be relatively non-reactive and thus thermodynamically stable, which is also supported by its large HOMO/LUMO gap and noticeable value of its chemical potential, −3.78 eV. 3) Furthermore, the global softness of the compound has low value, 0.146 eV, which supports the relatively low reactivity of the compound, and global electrophilicity of the compound can be considered as relatively noticeable, 2.083 eV, which would imply that this compound might show electrophilic behavior in chemical processes.

**TABLE 9:** Calculated GRPs for the isomer 5 (*eV*).

*IP*	*EA*	*Gap*	*X*	*η*	*Μ*	*σ*	*ω*
7.21	0.35	6.86	3.78	3.43	−3.78	0.146	2.083

## 4 Discussion

The original rule of five (Lipinski’s RO5) dealt with orally active compounds and had defined four simple physicochemical parameters, i.e., molecular weight ≤ 500, log *p* ≤ 5 representing hydrophobicity, H-bond donors ≤5, and H-bond acceptors ≤10. These properties have been associated with 90% of the orally active drugs that have achieved phase II clinical status (Lipinski, 2004). 4-Hydroxyisoleucine is compliant with one of the most acceptable drug-like parameters. Passing RO5 means acceptable aqueous solubility along with intestinal permeability, which are the first steps in oral bioavailability. The RO5 has found its importance where medicinal and combinatorial chemistry produced thousands of compounds with very poor physicochemical properties. However, passing RO5 never guaranteed that a compound will be drug-like. But if a compound fails, then a high probability of oral activity problems exists.

The molecular weight of 4-hydroxyisoleucine is 147.09, which is ideal for oral drug designing. The intrinsic molecular volume is considered as a molecular descriptor in modeling physicochemical properties and the biological activity. The molecular volume is one of the most common descriptors in quantitative structure–activity relationship (QSAR) studies (Leahy, 1986; McGowan and Mellors, 1986; Consonni et al., 2002; Zhao et al., 2003a). It is related to different physicochemical properties and biological processes including intestinal absorption and BBB penetration, to name a few (Zhao et al., 2003b). The number of hydrogen bond acceptors and donors are within the required strict limits. Oral drugs usually have fewer H-bond acceptors, H-bond donors, and rotatable bonds (Bitew et al., 2021).

The parameter of rotatable bond count is used as “drug filter.” More than 10 rotatable bonds are associated with decreased rat oral bioavailability (Veber et al., 2002). The mechanistic basis for this “rotatable bond filter” is not clear, since its count does not relate to the *in vivo* clearance rate in rats. However, the filter still remains reasonable from a viewpoint of *in vitro* screening as ligand affinity decreases at an average of 0.5 kcal for each two rotatable bonds (Dashti et al., 2014). The number of aromatic and heteroaromatic rings in a molecule has a relation with aqueous solubility, lipophilicity, oral bioavailability, serum albumin binding, CYP450 inhibition, and hERG inhibition. The fewer the aromatic rings in an oral drug candidate, the greater is the developable probability of a drug candidate. However, >3 aromatic rings in a molecule are linked with poorer compound developability and an increased risk of knock out. Even within the defined lipophilicity range, increasing aromatic rings lead to decreased aqueous solubility (Ritchie and Macdonald, 2009).

Saturated heterocycles are metabolized at the position of heteroatom or adjacent to it (Jean and Fotsch, 2012). The heteroatom is sometimes beneficial in drug metabolism like nitrogen in 4-hydroxyisoleucine. Redox reactions of drug metabolism such as heteroatom dealkylation, hydroxylation, heteroatom oxygenation, reduction, and dehydrogenation can yield active metabolites. Rarely, even conjugation reactions can give rise to an active metabolite (Obach, 2013). 4-Hydroxyisoleucine has a single rigid bond. This makes the molecule flexible enough to achieve a stable conformation at its target. The optimal range for drug-like property, TPSA, is 0–140 Å^2^, that of 4-Hydroxyisoleucine within accurate limit (83.55 Å^2^). Molecules with a TPSA of ≥140 Å^2^ would be poorly absorbed (<10% of the fraction absorbed), while a TPSA of 60 Å^2^ favors >90% absorption (Clark, 1999).

The calculation of QED score uses eight important properties that were previously used to assess drug-likeness, i.e., molecular weight, octanol–water partition coefficient (ALOGP), number of H-bond donors, number of H-bond acceptors, molecular polar surface area, number of rotatable bonds, number of aromatic rings, and number of structural alerts (ALERTS). ALERTS is a list of undesirable chemical features such as chemical reactivity or perceived toxicity. Drugs with high QED scores achieve higher absorption and bioavailability, have lower doses, fewer drug–drug interaction warnings and P-glycoprotein interactions, and lower absorption issues due to a food-related effect. Nonetheless, it is surprising that high QED-scoring drugs have similarities to low-scoring drugs with respect to plasma free fraction, the extent of gut-wall metabolism, first-pass hepatic extraction/metabolism, volume of distribution, clearance, elimination half-life, and frequency of dosing (Ritchie and Macdonald, 2014). In this context, 4-hydroxyisoleucine lies at the borderline of attractive and unattractive molecules. Thus, this negligible amount of difference may not provide an impetus for rejection.

Fsp^3^ is the fraction of sp^3^ carbon atoms. It is the number of sp^3^-hybridized carbons out of the total carbon count which tells about carbon saturation of molecules and characterizes the complexity of their spatial structure (Lovering et al., 2009). Natural products generally possess a higher fraction of sp^3^ than synthetic compounds and so become a rich source of drugs (Jia et al., 2020). Yan and Gesteiger (2003) used it to characterize aliphatic degrees of molecules and ultimately predict solubility (Yan and Gasteiger, 2003). The optimum value of ≥0.42 for Fsp^3^ is considered a suitable value, and about 84% of marketed drugs meet this criterion (Kombo et al., 2013). However, sp^3^ content should be increased within an appropriate range; higher Fsp^3^ score does not always assure higher performance and can increase the difficulty of synthesis (Gerlach et al., 2019). However, critical discussions of Fsp^3^ scoring have helped in generating new potential descriptors, such as MCE-18, which is discussed in the following paragraphs, and calculated using the following equation (Wei et al., 2020).

$$\text{MCE‐18}=\left(AR+NAR+CHIRAL+SPIRO+sp3+cyc-A_{cyc/1}+{sp}^{3}\right)\;x\;Q_{1}.$$

A simple sp^3^ index (number of sp^3^-hybridized carbon atoms) can lead to a high rate of false-positive results, while the issue can be solved with MCE-18 descriptor. The composition, spatial geometry, and complexity of chemical structures especially for state-of-the-art molecules are not considered in sp^3^ index, as these special features cannot be reflected using only the portion of sp^3^-hybridized carbons. They focus on the nature and quality of sp^3^-rich frameworks rather than sp^3^ counts only (Ivanenkov et al., 2019).

4-Hydroxyisoleucine is a naturally derived molecule, and therefore, MCE-18 may not be the only guide toward drug discovery. MCE-18 is applied for the assessment of pharmacological novelty and may help in designing new potential chemical entities in modern drug design. This index is also used for profiling HTS-focused libraries and prioritization of molecules. Presumably, compounds with optimal MCE-18 values have greater reliable IP positions. It is a tracer of medicinal chemistry evolution and is used to assess the “distance” between old and new chemistry. During the past 10 years, two of the top four pharmaceutical companies have primarily focused on molecules with MCE-18 values over 60, while the remaining two companies have demonstrated less drug productivity but have reached similar scores (Ivanenkov et al., 2019). The natural product score is congruent with the real-time natural source of 4-hydroxyisoleucine, namely, fenugreek seeds (Fowden et al., 1973).

4-Hydroxyisoleucine is compliant with the Lipinski rule, Pfizer rule, and GSK rule. The parameters for the Pfizer rule are logP >3 and TPSA <75. Compounds with higher log P (>3) and low TPSA (<75) are more likely to be toxic. The most significant work which describes the influence of molecular properties on toxicological outcomes has led to the “3/75 rule.” This has been devised by Pfizer from an analysis of exploratory or dose-finding toxicology studies of 245 compounds. This approach defined that compound with logP <3 and tPSA >75Å^2^ are expected to be non-toxic (Hughes et al., 2008; Waring et al., 2015). However, 4-hydroxyisoleucine is non-toxic and classified as safe with the Pfizer rule. The GSK rule suggests MW ≤ 400 and logP ≤4 (Hann, 2011) which are well qualified by 4-hydroxyisoleucine. The Golden Triangle aids in achieving metabolically stable and permeable drug candidates. It is a visualization tool developed with three parameters i.e., *in vitro* permeability, *in vitro* clearance, and computational data (Johnson et al., 2009). However, the stability and permeation of 4-hydroxyisoleucine is already demonstrated (Gowtham et al., 2022). Thus, the predicted Golden Triangle rejection may be ruled out here.

PAINS, ALARM NMR, BMS, and chelator rule are in favor of 4-hydroxyisoleucine. Pan assay interference compounds have a tendency to produce false-positive hits in high-throughput screening. The mechanism of PAINS activity is poorly understood. However, PAINS are associated with protein reactivity and non-covalent interactions (Bolz et al., 2021). ALARM NMR is critical to recognize reactive compounds that should be prioritized for lead optimization. This has emerged as a new filtering tool that can identify nuisance compounds and ultimately improve the process of hit triage. It is a La assay to detect reactive molecules by NMR by monitoring dithiothreitol-dependent ^13^C chemical shift changes brought in the human La antigen (Huth et al., 2005; Zega, 2017; Dahlin et al., 2018). Thiol reactive compounds oxidize or form covalent adducts with thiol groups of proteins. Therefore, ALARM NMR is a sensitive tool to rapidly identify compounds with increased risk of side effects in humans, such as alcohol intolerance, reactive oxygen species generation, and drug–drug interactions (Huth et al., 2007). The BMS is an alert for mapping molecular promiscuity and identification of undesirable and reactive compounds, while the chelator rule reflects chelating compounds (Agrawal et al., 2010; Yang et al., 2021).

Caco-2 cell lines are still widely used in absorption studies. Sometime regarded as the “gold standard” technique, Caco-2 cells are used to validate other absorption assays (Artursson et al., 2001; Hidalgo, 2001; Ding et al., 2021; Murador et al., 2021). 4-Hydroxyisoleucine has a lower Caco-2 cell-predicted permeability. However, a considerable chance of MDCK permeability exists. The MDCK cell line is also used for permeability screening in drug discovery (BOKULIĆ et al., 2022). However, both ADMETLab 2.0 and SwissADME are in favor of better gastrointestinal absorption and bioavailability, indicated by the boiled-egg plot as well. However, there are no permeability results available from wet labs. 4-Hydroxyisoleucine is safe from clinically significant drug interactions. The absorption and excretion of Pgp substrate can be modified by its inducer/inhibitor, ultimately paving the way for drug interaction (Yamazaki et al., 2019). A Pgp inhibitor is more likely to have interactions (Telbisz et al., 2021; Terrier et al., 2021). ADMETLab 2.0 and SwissADME declared it as a non-Pgp substrate, diminishing the risk of potential drug interactions and drug resistance mechanisms. Metabolic enzyme inducers or inhibitors can increase or decrease the blood level of another drug which is a substrate for the target enzyme. This can lead to several pharmacokinetic drug interactions with CYP inhibitors or inducers (Bolleddula, 2021; Lang et al., 2021). Only a marginal chance of being CYP2CP substrate is predicted for 4-hydroxyisoleucine. However, no other risk exists for a significant drug interaction. To date, there are no studies available on 4-hydroxyisoleucine–Pgp interactions. Studies on the metabolism of 4-hydroxy isoleucine are also desirable to establish a complete pharmacokinetic profile.

Some of the pharmacokinetic parameters of 4-hydroxyisoleucine (10 mg/kg) were estimated after oral administration in Wistar rats using the ultra-performance liquid chromatography–tandem mass spectrometry-based method. The V_d_ was estimated as 3,123.59 ± 355.86 mL/kg (Wadhwa et al., 2020), which is lower than that calculated by ADMETLab. The clearance of 4-hydroxyisoleucine appeared to be 204.95 ± 23.97 mL/h/kg which is less than the rate determined by ADMETLab. However, the t_1/2_ 10.83 ± 1.96 h is almost equivalent to that determined by ADMETLab (Wadhwa et al., 2020). The long half-life is congruent with the low clearance rate of 4-hydroxyisoleucine.

A drug with a moderate clearance rate and a better half-life may provide dose flexibility and better patient compliance (Sleep et al., 2013). Also, 4-hydroxy isoleucine is assumed to have a desirable clearance rate and half-life. However, a risk of CNS unwanted effects can be expected as 4-hydroxyisoleucine has the probability to be BBB permeant according to ADMETLab 2.0. Contrary to this, the boiled-egg plot predicted with SwissADME showed that it is non-permeant across the BBB (Daina and Zoete, 2016).

While evaluating toxicity, not even a single risk was found for hERG blockade, H-HT, DILI, Ames toxicity, rat oral acute toxicity, skin sensitization, carcinogenicity, and ophthalmic toxicity. Cardiac arrhythmia is one of the most frequently encountered side effects during drug discovery. This effect is related to inhibition of the hERG cardiac potassium channel. Also, this is the reason regulatory agencies such as US Food and Drug Administration (FDA) and European Medicines Agency recommend early evaluation of hERG toxicity (Garrido et al., 2020). Both ADMETLab 2.0 and Pred-hERG defined 4-hydroxyisoleucine as non-hERG blocker, being safe from cardiotoxicity. Hepatotoxicity is also highly responsible for withdrawal of an approved drug from the market. Hepatotoxicity accounts for >50% of acute liver failure cases as > 600 drugs are associated with it (Williams, 2018). Drug-induced liver injury is one of the leading factors of drug failure in clinical trials. Therefore, its assessment of drug candidates is necessary in advance as an effective strategy to decrease the drug attrition rate (He et al., 2019; Walker et al., 2020). The Ames toxicity test is an indicator for assessing mutagenic compounds’ potential and use bacteria for this assay. 4-HIL is likely to be safe from these types of toxicities (Petkov et al., 2021). However, the risk of respiratory toxicity has yet to be determined.

Toxicity studies on 4-hydroxyisoleucine are scant; however, studies on standardized extracts of its natural origin exist. Singh et al. (2022) evaluated the toxicity of 4-hydroxyisoleucine using human embryonic kidney cells (HEK-293) and healthy rats. The viability of HEK-293 cells was not affected by 4-hydroxyisoleucine, and no signs of toxicity were observed in healthy rats. Another study by Swaroop et al. identified *Trigonella foenum-graecum* seed extract (source of 4-Hydroxyisoleucine) as safe and efficacious. After extensive acute oral toxicity, 28 days’ sub-chronic toxicity and Ames’ bacterial reverse mutation assay, no toxicity was detected (Swaroop et al., 2014).

Deshpande et al. observed IDM01 (4-hydroxyisoleucine and trigonelline-based standardized fenugreek seed extract) as safe during acute and sub-chronic preclinical toxicity in rats without mutagenicity or genotoxicity. Not a single mortality or treatment-related adverse sign was noted during acute (≤2000 mg/kg) and sub-chronic (90-day repeated doses of 250/500/1,000 mg per kg body weight with 28 days of recovery period). The oral median lethal dose (LD_50_) was >2,000 mg/kg during the acute oral toxicity study. A non-observed adverse effect level was estimated at 500 mg/kg. The Ames test did not exhibit any mutagenicity up to 5,000 μg/plate and did not induce any structural chromosomal aberrations at < 50 mg/culture (Deshpande et al., 2017).

A genotoxicity study of the fenugreek seed extract (≤40% 4-hydroxyisoleucine) was carried out *via* standard battery of tests such as reverse mutation assay, mouse lymphoma forward mutation assay, and mouse micronucleus assay. There was no genotoxicity exhibited under the tested conditions (Flammang et al., 2004).

Bioconcentration represents the accumulation of chemicals in organisms at higher concentration than its surrounding (Jambor and Weisener, 2005). Bioconcentration factors are considered to assess secondary poisoning potential and risks to human health *via* the food chain. The factor is an estimate of the residual organic chemicals used for ranking chemicals as possible hazards to the environment (Gramatica and Papa, 2003). Similarly, the toxicity of organic aromatic compounds is based on logarithm of 50% growth inhibitory concentration (log (IGC_50_-1)) of *Tetrahymena pyriformis*, and fathead minnow which have extensive use in ecotoxicology and environmental safety applications (Keshavarz et al., 2021). The environmental toxicity of 4-hydroxyisoleucine exhibited a higher LC_50_, favoring the evidence of being non-ecotoxic. But the most acceptable evidence can be its origin as natural product, being part of nature chemistry (Avalos-Soriano et al., 2016). In the same manner, no risk of toxicity is predicted for androgen and estrogen receptors, PPAR-γ, heat shock element, mitochondrial membrane potential, and p53 (Bova et al., 2005; Ciocca et al., 2013; Khoo et al., 2014; Barna et al., 2018; Wahl and Smieško, 2018; Xi et al., 2020; Ciallella et al., 2021). Androgen-disruptors include all those chemicals which interfere with the biosynthesis, metabolism, or its physiological action. This results in an abnormal male development characteristic and poor growth and function of reproductive tract. Since androgen is the main regulator, its disruptors can harm reproductive developmental processes (Luccio-Camelo and Prins, 2011). In the same manner, estrogen disruptors can harm the female reproductive programming as the estrogen receptor is the primary determinant of female characteristics (Tsakovska et al., 2011). However, 4-hydroxyisoleucine is devoid of such toxicities and is considered non-toxic.

Mitochondrial membrane potential is generated by proton pumps and one of the essential components in the energy storage process. It is kept stable, and little variations are allowed; however, long-lasting disturbance may lead to adverse pathologies and loss of cell viability (Zorova et al., 2018). 4-Hydroxyisoleucine is free from such disruption of mitochondrial membrane potential. Similarly, genetic toxicology is important for long-term carcinogenicity during the early drug development process. This genotoxicity testing can identify the potentially hazardous drug candidates which may be knocked out at later stages of the drug development process (Custer and Sweder, 2008). However, ADMETLab recommends 4-hydroxyisoleucine as non-carcinogenic and non-genotoxic.

As all these parameters are modifiers of normal human physiology, and their disturbance leads to a red alert toxicity. Furthermore, none of the toxicophores were found in 4-hydroxyisoleucine which may have led to aquatic toxicity, skin sensitization, or created an acute toxicity response. The molecule has proven to be biodegradable and is not enlisted in the SureChEMBL database, implying medicinal chemistry friendly status (Papadatos et al., 2016; Falaguera and Mestres, 2021). The DFT study results provide certain support for the aforementioned discussion. Thus, formation of intra- and intermolecular hydrogen bonds by the 4-hydroxyisoleucine molecules, along with negative and positive MEP accumulation and significant NBO charges, would imply strong interactions with polar solvent molecules, including water, and thus acceptable aqueous solubility of the compound. Also, this would imply interaction of the 4-hydroxyisoleucine molecules with polar parts of large organic molecules. Furthermore, the shown presence of a few H-bond donors and acceptors would support the suggestion of the potential of the compound as an oral drug. Moreover, formation of several low-lying structural isomers confirms that the molecule is flexible enough to achieve a stable conformation at its target. The 4-hydroxyisoleucine stability shown by the GRP analysis supports its long half-life and stability findings achieved by other approaches.

## 5 Conclusion

To determine the knockout of 4-hydroxyisoleucine, a detailed ADMET profile was predicted *via* the ADMETLab 2.0 database. This molecule has satisfactory physicochemical properties and medicinal chemistry. The pharmacokinetic profile is in the favor of oral bioavailability, efficient distribution to all tissues including the brain, and non-interruptive drug metabolism, with moderate clearance and half-life. Some of the actual PK parameters are almost comparable to *in silico* values, but further detailed studies are desirable in the lab. There is no risk of any toxicity predicted both *in silico* and inside lab, except with low probability of respiratory toxicity predicted. Even it is undesirable for this molecule to disturb some of the most important receptors/targets such as gonadal receptors, p53, heat shock factor, mitochondrial membrane potential, or PPAR-γ. The molecule is safe for the environment, with agreeable lethal concentrations for some of the most common indicators. Finally, not a single toxicophore alert was found for 4-hydroxyisoleucine. All these properties suggest the drug ability of 4-hydroxy isoleucine. DFT study results are in support of the findings obtained by other methods.

## Data Availability

The original contributions presented in the study are included in the article/Supplementary Material; further inquiries can be directed to the corresponding author.
